# Yellow fever in Brazil threatens successful recovery of endangered golden lion tamarins

**DOI:** 10.1038/s41598-019-49199-6

**Published:** 2019-09-10

**Authors:** James M. Dietz, Sarah J. Hankerson, Brenda Rocha Alexandre, Malinda D. Henry, Andréia F. Martins, Luís Paulo Ferraz, Carlos R. Ruiz-Miranda

**Affiliations:** 1 Save the Golden Lion Tamarin, Silver Spring, Maryland, 22842 USA; 2 Associação Mico-Leão-Dourado, Casimiro de Abreu, CP 109968, CEP 28860-970, Rio de Janeiro, Brazil; 3 Department of Psychology, University of St. Thomas, St. Paul, Minnesota, 55403 USA; 4 Instituto de Geociências, Universidade Federal Fluminense, Campus Praia Vermelha, Niterói, Rio de Janeiro CEP 24210-240 Brazil; 5 Instituto de Biodiversidade e Sustentabilidade (NUPEM/UFRJ), Universidade Federal do Rio de Janeiro, Avenida São José do Barreto 764, São José do Barreto, Macaé, CEP 27965-045, Rio de Janeiro, Brazil; 6 Laboratório de Ciências Ambientais, Universidade Estadual do Norte Fluminense, Campos dos Goytacazes, CEP 28013-602 Rio de Janeiro, Brazil

**Keywords:** Conservation biology, Viral infection

## Abstract

The golden lion tamarin is an endangered primate endemic to Brazil’s Atlantic Forest. Centuries of deforestation reduced numbers to a few hundred individuals in isolated forest fragments 80 km from Rio de Janeiro city. Intensive conservation action including reintroduction of zoo-born tamarins into forest fragments 1984–2000, increased numbers to about 3,700 in 2014. Beginning in November 2016, southeastern Brazil experienced the most severe yellow fever epidemic/epizootic in the country in 80 years. In May 2018, we documented the first death of a golden lion tamarin due to yellow fever. We re-evaluated population sizes and compared them to results of a census completed in 2014. Tamarin numbers declined 32%, with ca. 2,516 individuals remaining *in situ*. Tamarin losses were significantly greater in forest fragments that were larger, had less forest edge and had better forest connectivity, factors that may favor the mosquito vectors of yellow fever. The future of golden lion tamarins depends on the extent of additional mortality, whether some tamarins survive the disease and acquire immunity, and the potential development of a vaccine to protect the species against yellow fever.

## Introduction

**Figure 1:**
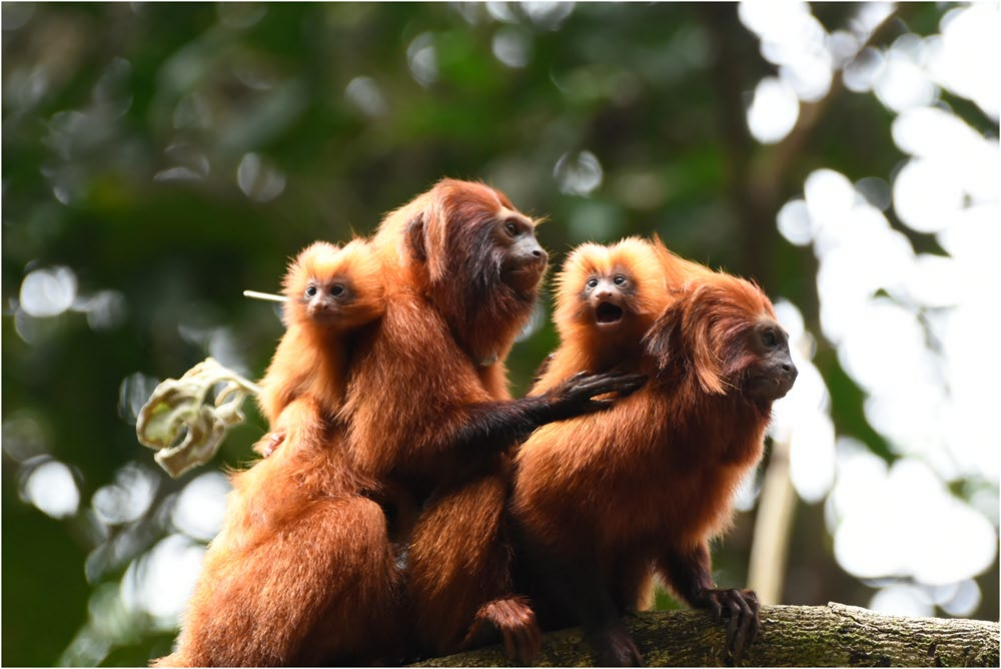
A family group of golden lion tamarins in Brazil’s Atlantic Forest, Rio de Janeiro State. Associação Mico-Leão-Dourado monitors about 15 groups of tamarins to detect changes in population sizes.

Golden lion tamarins (*Leontopithecus rosalia*; GLTs) are arboreal primates weighing ca. 600 g (see Fig. 1). Their pelage is golden-orange and they have a distinctive lion-like mane. The historical distribution of GLTs once included the Atlantic coastal forests of southern Espírito Santo state and Rio de Janeiro state (formerly Guanabara), southeastern Brazil^1^. Centuries of habitat destruction and capture of GLTs for sale to zoos and the pet trade combined to reduce the species to near extinction. In 1971, fewer than 400 individuals were thought to remain in the wild^1,2^. Early research reported that the range of GLTs was limited to five municipalities in the São João river basin in Rio de Janeiro state, 80 km northeast of the city of Rio de Janeiro, which contained over 12 million human inhabitants^1^. In this report we summarize the conservation work that resulted in the recovery of golden lion tamarin populations and we document a new threat to the species *in situ*, a severe outbreak of yellow fever in southeastern Brazil. We report the results of a survey to estimate GLT losses to yellow fever, identify environmental variables that explain geographic variation in GLT losses, and we make recommendations for the conservation of GLTs in the face of this disease.

The recovery of GLT populations in captivity and in the wild is seen as a conservation victory^3–5^. In the 1980s, research on captive breeding of GLTs improved survival and reproduction *ex situ*^6^. The number of GLTs and holding institutions increased to ca. 500 and 150, respectively, with the management goal of maintaining 90% of the genetic diversity of native GLTs^6,7^. While captive breeding of GLTs was seen as a model for recovery of an endangered species, habitat loss and degradation continued unabated in Rio de Janeiro state, Brazil. In 1983, the Smithsonian Institution’s U.S. National Zoological Park and Brazilian partners initiated the Golden Lion Tamarin Conservation Program, initially consisting of long-term field research on GLTs in Poço das Antas Reserve and community education using GLTs as a flagship species for habitat preservation^8–11^. When information on the biology of wild GLTs became available [e.g.^12–14^], program staff and partners held workshops to set and refine conservation goals with a desired outcome of an *in-situ* GLT population with 0% probability of extinction and 98% retention of genetic diversity over a period of 100 years as modeled using VORTEX simulation software (version 9.99)^15–17^. Results from the modeling indicated that at least 1,000 GLTs would be necessary to meet those demographic and genetic criteria^18,19^. The goal was increased to 2,000 GLTs in at least 25,000 ha of connected and protected forest as a buffer against future loss of forest. These science-based targets were adopted as conservation goals for the species in 2005.

**Figure 2:**
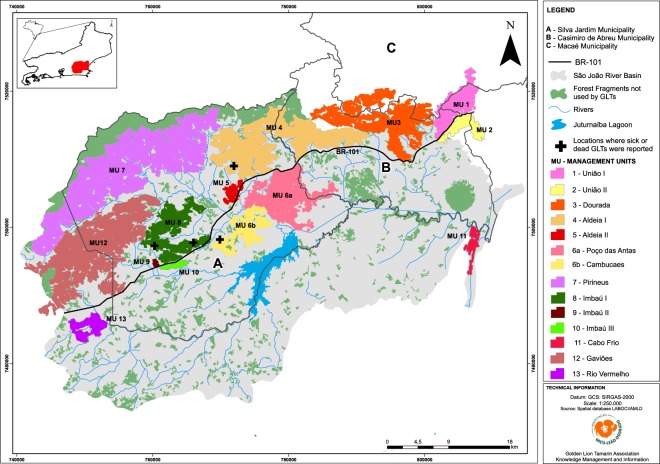
São João river basin, geographic range of most golden lion tamarins, 80 km northeast of the city of Rio de Janeiro, Brazil. Management units (MUs) are fragments of forest used by tamarins (i.e. below 500 m elevation) that are partially or completely isolated from other fragments of tamarin habitat.

From 1984–2001, program staff and assistants from the local community reintroduced 146 zoo-born GLTs, the majority on 40 private properties in the São João river basin, Silva Jardim Municipality (Fig. 2)^4^. Reintroduced GLTs were chosen from 43 institutions in 8 countries with the objectives of reintroducing a significant portion of the genetic diversity represented in the captive population and increasing numbers in the wild^19^.

Descendants of reintroduced GLTs flourished^20^ and increased to ca. 1,100 individuals. From 1994–1998, 42 native GLTs, i.e., animals not descended from zoo-born individuals were rescued from small forest fragments and translocated to a large fragment that would become the União Biological Reserve, Rio de Janeiro state. Descendants of these translocated GLTs, ca. 236 individuals, occupied all suitable habitat in that forest fragment^4,5,21^. In 1992, the U.S. National Zoological Park’s GLT Conservation Program was transformed into the Associação Mico-Leão-Dourado (AMLD; the golden lion tamarin association), a Brazilian non-governmental organization with the mission of achieving the science-based conservation goals of 2,000 GLTs in 25,000 ha of connected and protected forest. In 2003, the IUCN conservation status of GLTs was changed from “critically endangered” to “endangered”^22^. In 2014, AMLD completed a census of GLTs throughout their geographic distribution. Results indicated that ca. 3,706 GLTs lived in 41,411 ha of forest, albeit not all connected and protected^5^. GLTs were found in 13 forest fragments, which we refer to as management units (MUs), that are completely or partially isolated from one another (Fig. 2). The lack of habitat connectivity results in reduced gene flow among GLT populations as evidenced by inbreeding and loss of alleles reported in 2017^23,24^.

The yellow fever virus is endemic to regions of Africa and the Americas^25–28^. It is transmitted to humans or non-human primates through the bite of infected mosquitoes such as those in the genera *Aedes*, *Haemogogus* and *Sabethes*^29^. All Brazilian primates are susceptible to it^30^, including *Callithrix*, *Leontopithecus*, *Sapajus* and *Alouatta*, the four genera co-occurring in the São João river basin. Three transmission cycles are recognized for the virus: sylvatic, savannah (intermediate) and urban^29^. In the sylvatic cycle, the virus is transmitted to non-human primates by infected mosquitoes. Some mosquitoes transmit the virus to their eggs^31^, thereby maintaining the virus in the absence of primates. Although GLTs typically do not cross areas of non-forest^32^, mosquito vectors of yellow fever can disperse several kilometers over non-forest terrain^33^. Increased travel of people to and through forests near urban areas in southeastern Brazil increases the risk of humans contracting yellow fever from infected mosquitoes and/or spreading the disease to non-human primates in peri-urban forests^34^. Most people infected with yellow fever experience mild or no symptoms^35^. A small minority of infected people develop a more serious form of the disease resulting in severe symptoms or death^36^. An effective vaccine provides life-long immunity to the disease in humans but no yellow fever vaccine exists for non-human primates^34^. The mortality rate in non-human primates infected with yellow fever virus varies with species. Mortality is high in howler monkeys (*Alouatta spp*.) and marmosets (*Callithrix spp*.), and intermediate in capuchin monkeys, (*Sapajus spp*.)^34^. Lack of howler monkey vocalizations and grouped temporal and spatial patterns of deaths have been a signal to local health authorities of the presence of the disease and the need to vaccinate people in the region^37–40^.

Beginning in November 2016, southeastern Brazil experienced the most severe yellow fever epidemic/epizootic in the country in 80 years^34^. As of April 2018, there were 1,833 reported cases and 578 deaths due to yellow fever in humans^34^. From July 2017 to May 2018, 752 non-human primate deaths were attributed to yellow fever in Rio de Janeiro state as confirmed by laboratory analyses using histopathologic, immunohistochemical or reverse transcription polymerase chain reaction techniques on deceased animals^41,42^. Relatively few monkeys that die in the forest are recovered and delivered to laboratories to determine cause of death, thus the number of confirmed deaths underestimates actual losses^38,43^, especially in small primates such as GLTs. Over 6,000 non-human primates are thought to have died of yellow fever in the states of São Paulo and Espírito Santo alone^38^.

## Results

### Yellow fever causes declines in GLT populations

The recent outbreak of yellow fever in Brazil began in December 2016 in Minas Gerais state and quickly spread to other states to the east and south^44^. Infections of humans and non-human primates showed a seasonal distribution with lowest numbers in June-September, the driest and coolest months in southeastern Brazil^44^. AMLD documented three episodes of yellow fever within the geographic distribution of GLTs in Rio de Janeiro state: November 2016–May 2017, October 2017–May 2018, and August 2018–April 2019 (Table 1). In the first episode, AMLD received reports of howler monkeys dying of yellow fever in Macaé municipality, the northern extreme of the distribution of GLTs in south-central Rio de Janeiro state^45^. By May 2017, human deaths were reported in the municipalities of Casimiro de Abreu, Macaé and Silva Jardim^46,47^. These three municipalities contain the majority of GLTs in the species’ geographic distribution (Fig. 2). AMLD did not detect mortality of GLTs during this period and it was unclear if GLTs were susceptible to the disease.

**Table 1:** Chronogram of observations related to yellow fever in the geographic distribution of golden lion tamarins, 2016–2019.

Date	Observation
November 2016–May 2017	Reports of dead howler monkeys in five locations in Macaé municipality. Two individuals tested positive for yellow fever.
March 2017	First death of a person caused by yellow fever in Rio de Janeiro state during this outbreak, Casimiro de Abreu municipality.
April–May 2017	Human deaths caused by yellow fever in Macaé and Silva Jardim municipalities.
October 2017, November 2017, January 2018, February 2018	Last observations of four groups of GLTs monitored by AMLD in Poço das Antas Biological Reserve (MU 6a). Subsequent efforts to locate these groups and four others with known territories were not successful.
April 2018	Report of a sick GLT in Aldeia I (MU 4). Remains of this individual were not recovered.
April 2018	Three recently dead howler monkeys found by AMLD in Poço das Antas Biological Reserve (MU 6a). Dead GLTs found in Imbaú I (MU 8) and Cambucaes (MU 6b).
May 2018	A second dead GLT found in Imbaú I (MU 8).
May 2018	Laboratory analysis confirms yellow fever in GLT found dead in Cambucaes (MU6b)^44^.
August 2018	AMLD recovered skeletons of four howler monkeys in Poço das Antas Biological Reserve (MU 6a).
October–December 2018	Deaths of at least 10 howler monkeys reported in a forest fragment in Casimiro de Abreu municipality^46^. One tested positive for yellow fever^46^.
April 2019	Three dead howler monkeys and two dead GLTs reported in Cambucaes (MU 6b). AMLD recovered part of the skeleton of one howler monkey. No remains of GLTs were found.

In the second episode, with the exception of one individual GLT (see discussion), eight complete groups disappeared from their territories in Poço das Antas Reserve (MU 6a). Four of these groups were actively monitored by AMLD and four were not monitored at the time but had known territories. The cause of the disappearance of ca. 39 individuals is unknown but yellow fever is a strong possibility (see discussion). The eight groups were part of AMLD’s GLT monitoring program, occupied stable territories and comprised individuals habituated to the presence of human observers. AMLD has monitored the compositions of 7–13 groups in this reserve at weekly intervals for over 30 years. Batteries in radiocollars on individuals in the four monitored groups failed in October–November 2017. After January 2018, AMLD biologists failed to find sign of these GLTs at bait platforms. Non-systematic surveys using recorded playbacks of GLT vocalizations also failed to detect the four monitored groups and the four groups not being monitored.

In April 2018, AMLD received a report of a GLT apparently sick, on the ground and unable to climb trees in Aldeia I (MU 4, Fig. 2). A day later AMLD field staff visited the location and were unable to find the animal. In April 2018, AMLD staff found three recently dead howler monkeys in Poço das Antas (MU 6a), a dead GLT in Cambucaes (MU 6b), and a dead GLT in Imbaú I (MU 8). In May 2018, AMLD staff found a second dead GLT in Imbaú I (MU 8). AMLD delivered the three dead GLTs to a Rio de Janeiro state health department laboratory. Cause of death was not determined for two GLTs. In May 2018, the laboratory determined that the GLT found in Cambucaes had been infected with yellow fever virus (MU 6b)^48^. This was the first documented case of a GLT dying of yellow fever. In the third episode of yellow fever, AMLD recovered skeletons of four more howler monkeys in Poço das Antas (MU 6a, 29 August 2018)^49^ and received reports of dead howler monkeys in a forest fragment in Casimiro de Abreu municipality^50^. One of the howler monkeys from Casimiro de Abreu tested positive for yellow fever^50^. In April 2019, a landowner reported two dead GLTs and three dead howler monkeys in Cambucaes (MU 6b). AMLD recovered part of the skeleton of one howler monkey but found no sign of the dead GLTs.

### GLT population size estimates, 2014 and 2018

**Figure 3:**
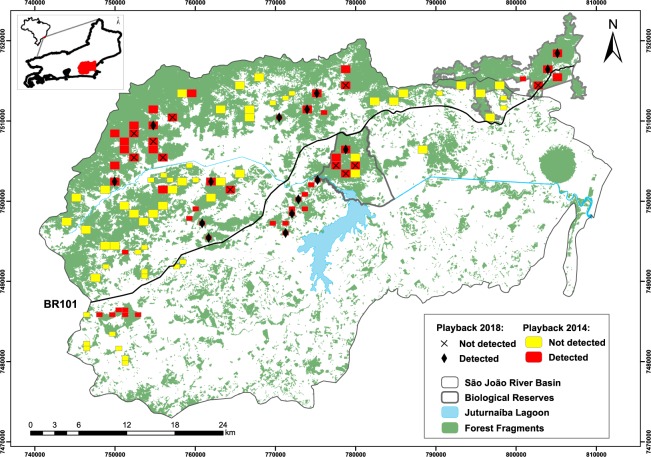
Results of the 2018 census of GLTs. Forest fragments (green) were identified using Landsat satellite images. Yellow and red polygons indicate randomly selected, 48 ha or 120 ha quadrats sampled in the 2014 playback survey. Quadrats resampled in 2018 are indicated by an X or a diamond. In 2014, observers played recorded GLT vocalizations at points 200 m apart along transects in each quadrat, noting responses by GLTs. In 2018, the distance between points was 100 m.

In 2013–2014, AMLD researchers used a modification of the point transect with lures method^51^ to estimate GLT population sizes in forest fragments throughout the species’ geographic distribution^5^. Methods included playing recorded GLT vocalizations along transects in 101 randomly selected quadrats and noting GLT responses. Given the pressing nature of the yellow-fever crisis, it was not possible to repeat the entire baseline survey completed in 2014. Instead, we resampled 26 quadrats sampled in the 2014 survey (Fig. 3). These quadrats were in six MUs estimated to contain ca. 90% of the total GLT population in 2014. In the 2014 survey, GLTs were detected in all 26 quadrats chosen for resampling in 2018. Partial results of the 2014 survey are presented in Table 2.

**Table 2:** Partial results of 2014 baseline playback survey (modified from^5^).

Management Unit (MU)	Forest area (ha)	Number quadrats sampled 2014	Quadrats with GLTs detected 2014	Percent quadrats with GLTs 2014	GLT population size estimates 2014	Number quadrats sampled 2018	Quadrats with GLTs detected 2018	Percent quadrats with GLTs 2018	GLT population size estimates 2018 Method 1	Assigned density (GLT/ha) 2018 Method 2	GLT population size estimates 2018 Method 2
4	6993	13	6	0.46	499*	4	3	0.75	438*	0.125	874*
6b	2524	10	10	1.00	351	4	4	1.00	411	0.144	363
8	3043	11	6	0.55	593*	4	3	0.75	520*	0.125	380*
6a	3450	7	5	0.71	380	4	1	0.25	111	0.0093	32
7	13451	24	15	0.63	1303	7	2	0.29	442	0.0093	125
1	2376	4	4	1.00	236**	3	2	0.67	185**	0.114	271**
TOTAL	31837	69	46		**3362**	26	15		**2107**		**2045**
95% CI					**1863–5383**				**1348–2845**		**1775–2317**

Four MUs sampled in 2014 were not sampled in 2018 and therefore were not included here. Results of the 2018 playback survey and GLT population size estimates calculated using methods 1 and 2. In method 1, GLT population estimates were calculated by multiplying percent of quadrats with GLTs detected in 2018 by the number of GLTs estimated for that MU in 2014. Population size estimates were then adjusted to account for possible false-negative detections. In method 2, GLT population estimates were calculated by multiplying the forest area of each MU by an assigned GLT density. Densities for four MUs were chosen from the range of densities reported from long-term research in Poço das Antas Reserve^48^ and on detection rates from the 2018 survey. Density estimates for Poço das Antas Reserve (MU6a) and Pirineus (MU7) were based on playback survey results and additional survey data collected in Poço das Antas in 2018. *Populations descended all or mostly from reintroduced zoo-born GLTs. **Population descended from translocated GLTs.

We used two methods to calculate numbers of GLTs in the six sampled MUs. Method one assumes that the percent reduction in quadrats with GLT detections in 2018, from the 100% detection rate in those quadrats in 2014, reflects a proportional reduction in the size of the GLT population in that MU as estimated in the 2014 baseline survey (Table 2). In method two (Table 2), we estimated population sizes by multiplying the amount of forest area in the MU by GLT densities that we assigned based on playback survey results and additional field observations (see methods). The overall reduction in number of GLTs in the six MUs sampled in both years is 37% using method one and 39% using method two (Table 2).

For two of the MUs not included in the 2018 survey (MUs 9 and 13), our monitoring data indicate that there was no reduction in GLT numbers since the 2014 survey (total estimated at 213 GLTs, all descendants of reintroduced zoo-born GLTs). We have no information on GLT numbers in the other five MUs and assumed no change in numbers since the 2014 survey, estimated at 258 GLTs (155 descendants of native GLTs, 41 descendants of reintroduced GLTs and 62 descendants of translocated GLTs). Adding the 471 GLTs in these seven MUs to the totals in Table 2 yields 2578 (method 1) and 2516, (method 2). These estimates represent an overall decline of 30% (method one) or 32% (method two) from the 3706 GLTs estimated *in situ* in the 2014 survey.

A comparison across the two surveys provides additional information supporting reductions in GLT populations since 2014. There was a significant change from 2014 to 2018 in the proportion of quadrats with detected GLTs (P < 0.001, N = 26). For 15 quadrats in which GLTs were detected in both surveys we calculated the number of meters along the transect to first detection of GLTs in 2014 and 2018. The number of meters to first detection was significantly greater in 2018 than in 2014 (t_14_ = 2.22, P = 0.044; 2014: Mean = 386.7, SE = 71.6; 2018: Mean = 667.0, SE = 92).

### Why were GLT declines greater in some MUs?

To explain the greater declines of GLTs in some areas compared to others we quantified seven landscape variables for the six sampled MUs (Table 3 and see methods). We ran a logistic regression to test for factors associated with changes in GLT detections in the 26 quadrats, 2014 and 2018. The initial model included the seven variables in Table 3, plus the number of native non-human primate species in the MU, and the presence/absence of non-native marmosets (*Callithrix spp*.). Three variables entered the final model: categorical ranking for forest area size in the MU, percent core area and dIIC, a measure of forest connectivity. The model was significant (χ^2^ = 9.70, P = 0.046, N = 26) and with these three variables correctly classified 72.7% of quadrats without GLTs and 80% of quadrats with GLTs. Quadrats with reduced detections of GLTs were more likely to be found within a larger MU with higher percent core area and higher dIIC.

**Table 3:** Estimates of seven landscape variables in the six sampled management units (MUs).

Management Unit (MU)	Forest area (ha)	Percent core area (%)	Elevation (m)	Distance to urban area (m)	Distance to paved road (m)	Distance to swamp (m)	Connectivity importance value (dIIC)
4	6993	54	255	3399	4025	9335	89.791
6b	1055	31	7	6887	3538	3211	70.993
8	3042	54	171	3699	3622	13011	72.487
6a	3450	64	46	8858	3703	2610	83.429
7	13450	66	29	3389	12400	20760	96.262
1	2247	71	121	205	1707	30040	68.171

## Discussion

This report documents the first death of a golden lion tamarin to yellow fever and the results of a survey quantifying GLT losses throughout the species’ geographic range. Despite documented concurrent yellow-fever-caused mortality in humans and two species of non-human primates (GLT and howler monkeys) in the municipalities where we conducted the 2018 survey, we cannot confirm that reductions in GLT numbers were caused by yellow fever alone. We considered three alternative explanations for the declines.

One alternative explanation is that GLTs were present in the forest fragments but were not detected during the 2018 playback survey. Prior to the 2014 baseline survey, AMLD researchers conducted experiments to determine the probability of response to a playback and the maximum distance from the playback speaker that would provoke a response from GLTs^5,52^. Results showed that GLTs respond to playbacks by vocalizing and approaching closely in over 80% of playbacks and that responses dropped off significantly at 120 m from the playback speaker. The distance between the playback points for the 2014 survey was 200 m. To reduce the possibility of not detecting a GLT group, in 2018 we did playbacks at intervals of 100 m. It is unlikely that trained observers would fail to detect GLTs at this distance. In addition, population estimates for 2018 were increased by 17% to account for potential false negative detections (lack of response by GLT present in the sampled quadrat).

A second alternative explanation for GLT reductions is that GLT populations were declining between 2014 and 2018 because of reduced birth rates or other demographic changes. To examine this possibility, we ran an analysis of variance comparing the compositions of 36 monitored groups for 2013–2014 (average of two years) with 19 groups monitored in 2018, after the onset of yellow fever in 2017. Group size, number of adults, number of subadults, number of juveniles and number of infants present in the groups were included as dependent variables. MU size was not a significant predictor of the dependent variables. Groups were not significantly larger in 2018 (P = 0.070) but contained significantly more adults (P = 0.015) and significantly fewer infants (P = 0.017) than in 2013–2014. It is important to note that GLTs that were monitored in late 2018 either survived yellow fever or were not exposed to the disease. Differentiating between these two circumstances will be important for evaluating future risk imposed by yellow fever and formulating strategies in response.

A third alternative explanation is that a mortality factor other than YF caused the reductions. Predation has been identified as the cause of significant changes in population demography in GLTs^53^ and other primate taxa^54,55^. In a study that has continued for over 35 years, AMLD researchers continuously monitor 7–13 groups of GLTs in Poço das Antas Reserve. GLTs in these groups are habituated to human observers and are individually tattooed and dye-marked. One or more individuals in each group carries radio-collars to facilitate detection. At weekly intervals, all births, deaths, emigrations and immigrations are recorded as is geographic location of the group. During 19 years of that study AMLD researchers documented an eight-year period of unprecedented high predation on GLTs in Poço das Antas Reserve (low predation: 1987–1995 and 2004–2005; high predation: 1996–2003). Tayras (*Eira barbara*) and perhaps other predators took GLTs at their den sites at night, often killing most individuals in the group. This period of high predation resulted in the largest documented reduction in a GLT population^53,56,57^. Mean group size dropped from 6.0 to 4.4, and mean density from 0.121 GLTs/ha to 0.109. Despite persistent high predation during these years, GLTs continued to occupy all suitable habitat in the study area and maintained a neighborhood of adjacent territories, a pattern very different from the vacant territories observed in 2018. It’s unlikely that the rapid losses of GLTs in 2018 is explained by predation.

Although hunting is illegal, it occurs occasionally in all MUs. However, it’s unlikely that hunting or poaching explain the declines in GLT populations. Hunters in this region hunt large mammals as a source of food. GLTs are not large enough to warrant shooting. The two biological reserves are patrolled by guards and AMLD field staff systematically monitor GLTs there and in several other MUs. Trapping GLTs requires setting bait platforms well in advance of trapping to habituate the tamarins to the presence of bait and traps. AMLD field staff would likely discover any attempt to trap GLTs in areas where GLTs are monitored using radiotelemetry. AMLD staff and members live in the local communities and actively support GLT conservation. Since the early 1980s, when AMLD began intensive environmental education in the region we have not observed poachers attempting to trap GLTs.

While we cannot rule out other diseases, yellow fever is the mortality factor most likely to have caused the reductions in GLT populations in 2018. We reach this conclusion because of the rapid and widespread disappearances of GLTs, the positive laboratory diagnostic testing for yellow fever in one dead GLT^48^, and the temporal and spatial coincidence with deaths of howler monkeys (*Alouatta guariba clamitans)*^49^, some confirmed to have been caused by yellow fever. Howler monkeys are extremely susceptible to the yellow fever virus and act as sentinels to its presence^30,58^. During January–April 2018, eight groups of GLTs disappeared from their territories in the Poço das Antas Reserve. From October to December 2018, AMLD researchers saw only 5 GLT groups in that Reserve. Group sizes were 4 (2 adults and 2 juveniles, the only GLTs detected in this MU in the 2018 playback survey, perhaps members of a former study group), 1 lone adult male, 5 (group composition not known), 4 (3 adults and one infant), and 2 adults. We compared these group sizes with those from eight groups in the Reserve before the outbreak of yellow fever: 2015 mean group size (adults only) = 4.88, SE = 0.789; 2018 mean = 2.60, SE = 0.678; t_11_ = 1.99, p = 0.072. In 2018, only one infant was seen in these groups during a period when all breeding groups typically carry new infants, usually twins^59^. The lone adult male spent eight months moving around the Reserve, apparently looking for a mate, and disappeared in 2019. We conclude that yellow fever is the most likely explanation for the near-complete decimation of the GLT population in Poço das Antas Reserve.

Although yellow fever has been documented in Rio de Janeiro since 1849, and there have been 19 outbreaks, each with at least 1,000 human deaths in Rio de Janeiro city^60^, the historical record provides little information about how previous outbreaks of yellow fever affected GLT populations. Surveillance of epizootics in non-human primates became a priority for Brazilian health authorities only in1999, and required reporting of deaths of non-human primates that might be related to human epidemics was required only in 2006^40^. Results of the first systematic census of GLTs throughout their geographic distribution was published in 2019^5^. When we began field research on GLTs in 1984, several forest fragments of suitable habitat did not contain GLTs. It’s impossible to know if GLTs in these fragments were decimated by a disease such as yellow fever or trapped for sale to legal or illegal animal trade markets.

Early studies on susceptibility to yellow fever virus (YFV) in non-human primate taxa currently in the geographic distribution of GLTs provide insight into potential effects of a yellow fever epizootic. In one study the researcher inoculated 14 GLTs with two Brazilian strains of YFV^61^. All individuals showed the virus circulating in their blood streams from days 2–6, and all succumbed. He also inoculated 137 common marmosets (*Callithrix jacchus*) with four Brazilian strains of YFV. Of these individuals, 133 either died or developed immunity. Mortality ranged from 50–91% and average survival time was about 8 days. Mosquitoes that fed on infected marmosets were capable of transmitting the virus to other marmosets. Surviving individuals acquired immunity^61^. In another study researchers inoculated 17 buffy-tufted marmosets (*Callithrix aurita)* with Brazilian strains of YFV^62^. Of these, 15 individuals died between days 4–7 and two survivors acquired immunity to the virus. These authors also exposed 8 black-tufted marmosets (*Callithrix penicillata)* to mosquitoes infected with YFV. All became infected, 6 died and the 2 survivors acquired immunity^62^. The results from studies on howler monkeys are similar: these monkeys are highly susceptible to YFV, infect mosquitoes readily and the few survivors acquire immunity^63^. In a series of experiments on five species of capuchin monkeys (*Cebus* and *Sapajus*), researchers found YFV circulating in the bloodstreams of inoculated individuals but subjects had a relatively low percent mortality^64–66^. Based on these early reports, Strode *et al.*^63^, concluded that *Haemagogus* mosquitoes are capable of rapidly infecting local populations of non-human primates which would result in explosive epizootics followed by near extinctions. The few survivors are assumed to acquire immunity to yellow fever for life.

Recent outbreaks of yellow fever are responsible for large-scale mortality in South American non-human primates. In 2007–2008, yellow fever outbreaks caused significant losses of howler monkeys in northeastern Argentina^67^. In 2009, yellow fever was implicated in the deaths of many howler monkeys in Rio Grande do Sul state, Brazil^68^. From 2007–2009, the Brazilian Ministry of Health received reports of 1971 localized epizootic outbreaks suspected to have been caused by yellow fever. Yellow fever was confirmed in 209 of the 3602 non-human primates involved. Of those non-human primates identified to genus, 64% were howler monkeys, 29% were marmosets (*Callithrix sp*.) and 7% were capuchin monkeys^69^. In 2016–2017, yellow fever was implicated in 10% and 26% reductions in two populations of northern muriqui (*Brachyteles hypoxanthus*) in Minas Gerais state, Brazil^70^.

Several hypotheses have been put forth to explain the prevalence of yellow fever outbreaks in relatively large geographic regions, e.g. continents, countries and states. Examples include climatic variables^71,72^, distribution of non-human primates^71,72^, altitude^72^ and proximity between urban and forest environments^34^. The geographic scope of our study is modest in comparison, an area of only 72 km × 20 km. To attempt to explain variation in losses of GLTs in this relatively small area we looked for relationships with landscape variables related to proximity of humans, the distribution of non-human primate species which may facilitate persistence of the virus in the forest, and environmental factors perhaps favoring survival and reproduction of mosquito vectors e.g. proximity to swamps. We found no statistical relationship between human activities and losses of GLTs to yellow fever. Proximity to roads or cities were not significant predictors of GLT losses in forest fragments. However, the forests in this region are heavily occupied by permanent and weekend residents living in villages, farms and ranches. Illegal hunting is common on some privately-owned land. Perhaps the highest human density is in Imbaú I (MU 8), an area that had relatively low losses of GLTs. The areas with the lowest human densities are União and Poço das Antas Reserves, which restrict access by people. União Reserve had relatively low losses of GLTs while Poço das Antas suffered very high losses.

Likewise, we found no relationship between the distribution of non-human primate species and GLTs losses. Decade-old surveys of non-human primates in the area show that non-native marmosets occur at moderate to high densities in all fragments where GLTs occur except the two biological reserves, Poço das Antas and União^73^. Howler and capuchin monkeys are absent from most smaller fragments and are present in the two biological reserves^74^. We did not attempt to quantify the density of non-human primates in the sampled areas. However, anecdotal observations suggest that areas of apparent high density of non-human primates, e.g. Imbaú I, did not have high rates of GLT losses. Pirineus (MU 7), an area with apparent low density of non-human primates, had high losses of GLTs.

The observed differences in GLT losses may be explained by environmental requirements of mosquito species that spread the virus. H*aemagogus* and *Sabethes* mosquitoes spread yellow fever in our region, with *Haemagogus* the most common vector^75–77^. These mosquitoes are known to lay their eggs in tree holes and bamboo^78^, especially in higher forest strata^75,79^. Relative humidity is known to influence their abundance^80^. Tree holes are important den sites for GLTs^56^ and are often located in trees with larger diameters^81^. Trees large enough to provide tree-hole dens and forest with higher strata typically are found in areas with taller and more mature forest, e.g. Poço das Antas and Pirineus. The variables that showed significant statistical relationships with GLT losses were forest area, percent core area and connectivity importance value, i.e., forest connectivity. We hypothesize that forest fragments that are larger, with less edge and higher intra-patch connectivity maintain consistently higher relative humidity, which favors abundance and reproduction of mosquitoes that transmit yellow fever^82^. In contrast, forests that are smaller and more fragmented are subject to greater edge effect, which may decrease relative humidity^83^ and micro-climate buffering^84^ and thereby decrease mosquito abundance, especially during the dry season.

In a recent study the primary vectors of the current sylvatic yellow fever outbreak were identified as *Haemagogus leucocelaenus* and *Hg*. *janthinomys*, both of which were found present during surveys in municipalities occupied by GLTs (Silva Jardim, Casimiro de Abreu and Macaé; Fig. 2)^77^. Surveys were conducted in 2016, before the yellow fever outbreak and in 2017–2018, during the outbreak. In Macaé, only one of six pools of *Hg*. *janthinomys* tested positive for the virus. Samples from mosquitoes in the other two municipalities tested negative. However, that study sampled only a single point each in Silva Jardim and Casimiro de Abreu. The small sample size may have contributed to non-detection of YFV in mosquitoes sampled in these areas. These results emphasize the need for more extensive monitoring of vector and virus presence and prevalence in order to better understand current and future risks to both human and non-human primate populations.

AMLD’s science-based strategic plan for conservation of GLTs is part of the Brazilian government’s plan for conservation of Atlantic Forest primates^85^ and includes planting native forest corridors to reconnect 13 forest fragments (MUs) and their GLT populations. Two thousand GLTs in connected forest are necessary to meet genetic and demographic goals for the species, 98% retention of genetic diversity and 0% probability of extinction during a period of 100 years^86^. Until forest corridors are established, management using translocated GLTs is necessary to maintain genetic diversity targets for GLT populations in the smaller forest fragments^4,5,19,87^. The number of GLTs remaining in the wild, estimated here at 2516 individuals (method 2), is just adequate to meet management goals. However, heavy losses of GLTs in what were two of the largest populations will make it much more difficult to reconnect forest fragments holding at least 2000 GLTs. Prior to the onset of yellow fever, connection of three MUs would meet management goals (MUs 7, 12, 8; Fig. 2). In light of current GLT population size estimates, it will be necessary to connect eight MUs (1, 3, 4, 7, 12, 8, 6b, 6a; Fig. 2). The cost of planting these additional forest corridors will be significant. For example, AMLD estimates the cost of completing planted forest connections between MUs 7 and 12 at US$138,216. Additional funds also will be necessary to monitor and manage small populations of GLTs by translocations until forest connections are in place.

Losses of GLTs in 2018 highlight the importance of conservation efforts in the 1980s and 1990s. Descendants of reintroduced zoo-born GLTs and translocated rescued native GLTs comprised ca. 41% of the *in-situ* population in 2014, and 57% (method 1) or 72% (method 2) in 2018. If AMLD and partners had not done reintroductions and translocations of GLTs in vacant habitat decades ago, we estimate that only 675 GLTs would remain in four MUs in 2018 (520 in the three MUs sampled (Table 2, method 2) plus 155 native GLTs in two MUs not sampled).

The future of conservation of GLTs depends on whether populations suffer additional losses to yellow fever in coming years. GLT populations have the potential to grow 13–14% per year^12,18,19^ and can quickly repopulate areas of adequate habitat if the mortality rate is not high. Continuous monitoring will be necessary to detect future losses to yellow fever and adapt strategies as appropriate. Additional research will be necessary to explain the differences in impact of yellow fever on GLT populations in MUs, and whether some GLTs survived the disease and acquired immunity to it. Finally, at present there is no vaccine to protect non-human primates from yellow fever. However, research to develop such a vaccine is underway^34^. If yellow fever persists in forests occupied by GLTs, a vaccine to protect GLTs may make the difference between losing this endangered species and keeping it from extinction.

## Methods

### Ethics approval and Brazilian research permits

All data were collected under research and ethics permit number 17409, issued to Associação Mico-Leão-Dourado. The permit was granted by Brazil’s Instituto Chico Mendes de Conservação da Biodiversidade (ICMBio), Sistema de Autorização e Informação em Biodiversidade (Sisbio), the federal agency responsible for authorizing research and conservation projects on Brazil’s biodiversity. Methods approved in this permit include playbacks of GLT vocalizations along designated transects and monitoring of GLT social groups using radio-telemetry. Delivery to a scientific institution of animals found dead does not require a permit under Brazilian law (Article 25, ICMBio Instrução Normativa No. 3, 1 September 2014).

### Estimating GLT population sizes based on 2014 and 2018 surveys using playbacks of GLT vocalizations

For the 2014 assessment^5^, areas to be sampled were selected by overlaying a grid of quadrats on a map of the forested area of the 13 MUs and randomly selecting 10% of the quadrats per MU. Quadrats were 48 ha (small quadrats) in forest fragments <10 km^2^ (small fragments) or 120 ha (large quadrats) in fragments >10 km^2^ (large fragments). The objective was to sample >20% of the area of each MU. In each of the 113 selected quadrats, researchers played GLT long call vocalizations at 200 m intervals and recorded responses from resident GLTs. Sampling continued along the transect until tamarins were detected or the maximum points for the quadrat size was reached. For large quadrats, up to10 points along the transect line were sampled. For small quadrats, up to 6 points along the line were sampled. The number of groups detected per sampled area was used to project the total number of groups in the MU. Average group size for known groups was used to estimate the abundance of GLTs in the MU. Playback methods for the 2014 census are detailed in Ruiz-Miranda *et al*.^5^.

In 2018, playback methods were similar to those used in the 2014 survey^5^ with one difference. Playbacks of GLT vocalizations along the transect line were conducted at 100 m intervals in 2018, and 200 m intervals in 2014. Playbacks continued until GLTs were detected or the researchers reached the end of the quadrat without detecting GLTs. In 2018, AMLD researchers played vocalizations at a maximum of 20 points along the transect in large quadrats and up to 12 points in small quadrats. While the average group size was taken from well-studied populations, observers noted the number of responding GLTs seen and heard, and age categories when possible. A total of 26 quadrats were selected for study in 2018, 8 small and 18 large. The same AMLD observers conducted both surveys and each person had over 15 yrs experience collecting data on GLTs.

We used two methods to estimate the number of GLTs in each sampled MU. In method 1 (Table 2), we multiplied the percent of quadrats in which GLTs were detected in 2018, representing reductions from the 100% detection rate in 2014, by the number of GLTs estimated for that MU in 2014. To adjust for the possibility of failing to detect GLTs in quadrats that were occupied by GLTs, we calculated the percent of quadrats sampled in 2014 in which we knew GLTs were present, but in which we failed to detect them using standardized methodology: 3 of 18 quadrats, or 17% false negative detections. To adjust for false negatives in 2018, we multiplied population estimates for each MU by 1.17.

In method 2 (Table 2), for each MU we multiplied its forested area by an assigned density of GLTs. For four MUs we assigned GLT densities based on the range of densities reported for long-term research in Poço das Antas Reserve^53^: 0.073–0.144 GLTs/ha. The densities chosen for each MU represent the best estimate given the percent of quadrats that were occupied in 2018. For MUs Aldeia I and Imbaú I we assigned the third-quartile density, 0.125 GLTs; for MU Cambucaes we assigned the maximum observed density, 0.144 GLTs/ha; for União I we assigned the average density reported for Poço das Antas, 0.114 GLTs/ha. Several observations in 2018 led us to believe that the density of GLTs in Poço das Antas was well below that reported by Hankerson and Dietz^53^. In January–April 2018, AMLD staff conducted playback surveys in the northern half of the Reserve and detected no GLTs in areas that were occupied previously by eight groups of GLTs. The low detection rate of 25% in the 2018 survey supported this conclusion. During extensive surveys along trails in the northern half of the Reserve from October–December 2018, AMLD observers saw only 16 GLTs in five groups in the northern half of the Reserve, with an average of 2.6 (SE = 0.678) adults per group. Thus, to estimate GLT density in Poço das Antas we divided 16 GLTs by half the forested area of the Reserve, 1725 hectares, which yielded 0.0093 GLTs/ha. AMLD does not monitor GLTs in MU Pirineus thus the only information we have on GLT density is from the 2014 and 2018 surveys. Because the 29% detection rate in 2018 was similar to that for Poço das Antas Reserve, 25%, we assigned the same density to both MUs.

#### Analysis of landscape variables

For each MU we assessed the values of seven landscape variables (Table 3). Percent core area was calculated in LS Metrics^88^, and connectivity importance value in Conefor 2.6^89^. Values for the other landscape variables were calculated in ArcGIS 10.5^90^. Five variables (area, percent core area, distance from urban areas, distance from swamp areas and connectivity importance) were based on maps produced by the Brazilian Foundation for Sustainable Development^91^ through visual interpretation of RapidEye satellite images collected between 2013 and 2015, with 5 m resolution. Distance from paved roads was based on a map produced by the Brazilian Institute of Geography and Statistics^92^ using a geographic ratio of 1:25000 (pixel size of ca.10 m). Elevation (resolution ca. 30 m) was derived from the digital elevation model (DEM) obtained from USGS National Elevation dataset^93^. All data were resampled to 5 m resolution for analysis.

We measured connectivity between forest patches using the integral index of connectivity (IIC)^53,94^. IIC is an index based on the concept of habitat availability, which quantifies inter-patch connectivity through graph structure (topological position) and intra-patch connectivity through habitat patch dimension (patch size)^88,95^. We computed IIC following^95^ as:

$$IIC=\mathop{\sum }\limits_{i=1}^{n}\mathop{\sum }\limits_{j=1}^{n}\frac{ai\ast aj}{1+nlij}/A{L}^{2}$$

where *ai* is the area of each habitat patch and *nlij* is the number of links in the shortest path between patches *i* and *j*, and *AL* is the total landscape area^94^. Calculating IIC requires two inputs of information: (1) the node attributes, i.e., area, and (2) the connection among each pair of nodes, which can be computed as distance^89^. Based on our experience following monitored groups of GLTs we assigned 100 m as the maximum distance that GLTs can cross through non-forest matrix^19^. In order to include the surrounding patches in the study design of the landscape analysis we assigned a 10 km diameter buffer from the centroid of each patch (MUs). GLTs can effectively disperse up to 8 km^87^ therefore a 10 km buffer is an appropriate size for this landscape.

We measured the importance of patch habitat for maintaining landscape connectivity using delta values in the integral index of connectivity (dIIC):

$$dIIC( \% )=\frac{I-I^{\prime} }{I}\times 100$$

where *I* is the index value when the landscape element is present in the landscape and *I’* is the index value after removal of that landscape element^94^. The connectivity importance value, dIIC, can be partitioned into three distinct fractions considering the different ways in which a landscape patch can contribute to habitat connectivity and availability in the landscape^96^:

$${\rm{dIIC}}={{\rm{dIIC}}}_{{\rm{intra}}}+{{\rm{dIIC}}}_{{\rm{flux}}}+{{\rm{dIIC}}}_{{\rm{connector}}}$$

where dIIC_intra_ corresponds to the patch contribution in the form of its area (intra-patch connectivity), dIIC_flux_ corresponds to the flux of dispersing organisms that move to or from the patch, and dIIC_connector_ corresponds to how much the individual patch contributes to connectivity between other patches by serving as a stepping stone. A high dIIC value implies that the loss of that patch would severely reduce the connectivity between it and other habitat patches^96^.

### Statistical analyses

We used a McNemar’s test to determine if there was a significant change in the number of quadrats with GLT detections between the 2014 and 2018 surveys. A mixed-model ANOVA was used to test the number of meters until first detection of GLTs between the two surveys. Year was used as a repeated-measures variable. MU size was included as a fixed, between-subjects variable. All statistical assumptions were met. All analyses were two-tailed.

We ran a logistic regression to determine which variables might impact the presence/absence of GLTs in the 26 surveyed quadrats. MU identity was included in the model to control for variance associated with MU-level data (e.g. population origin, management strategy and GLT habituation to presence of human observers). Potential explanatory variables included categorical rank (1–3) of the MU based on amount of forest area (ranked largest (3) to smallest (1): Pirineus and Aldeia I each ranked 3; Poço das Antas and Imbaú I each ranked 2; União I and Cambucaes each ranked 1), percent core area, mean elevation, distance from nearest urban area, distance from nearest paved road, distance from nearest swamp area, connectivity importance value (dIIC), number of non-human primate species in the MU, and presence/absence of non-native marmosets (*Callithrix spp*.). All statistical assumptions were met. The analysis was two-tailed.

## Data Availability

Data not included in this manuscript are available by contacting the corresponding author. To help ensure the safety of endangered golden lion tamarins we will not share geographic coordinates of tamarin group locations.
